# Molecular and clinicopathological characteristics of *ERBB2* gene fusions in 32,131 Chinese patients with solid tumors

**DOI:** 10.3389/fonc.2022.986674

**Published:** 2022-10-06

**Authors:** Yin Guan, Yutong Wang, Hongxia Li, Jing Meng, Xia You, Xiaofeng Zhu, Qin Zhang, Tingting Sun, Chuang Qi, Guangyu An, Ying Fan, Binghe Xu

**Affiliations:** ^1^ Department of Medical Oncology, Beijing Chao-Yang Hospital, Capital Medical University, Beijing, China; ^2^ Department of Oncology, Shengjing Hospital of China Medical University, Shenyang, China; ^3^ Department of Oncology, Shanxi Provincial People’s Hospital, Taiyuan, China; ^4^ Department of Medical Oncology, The Chinese People's Liberation Army (PLA) General Hospital, Beijing, China; ^5^ The Medical Department, Jiangsu Simcere Diagnostics Co., Ltd, Nanjing, China; ^6^ Medicial Department, Nanjing Simcere Medical Laboratory Science Co., Ltd, Nanjing, China; ^7^ The State Key Lab of Translational Medicine and Innovative Drug Development, Jiangsu Simcere Diagnostics Co., Ltd, Nanjing, China; ^8^ Department of Medical Oncology, National Cancer Center/National Clinical Research Center for Cancer/Cancer Hospital, Chinese Academy of Medical Sciences and Peking Union Medical College, Beijing, China

**Keywords:** next generation sequencing, *ERBB2* fusion, HER2, trastuzumab, clinical impact

## Abstract

*ERBB2* amplification is one of the most important and mature targets for HER2-targeted drug therapy. Somatic mutations of *ERBB2* in the tyrosine kinase domain have been studied extensively, and play a role in response to anti-HER2 therapy among different cancer types. However, *ERBB2* fusion has not been got attention and its relevance to HER2-targeted therapy is unclear. We comprehensively characterized *ERBB2* fusions from next-generation sequencing (NGS) data between May 2018 and October 2021 in 32,131 various solid tumors. Among the tumors, 0.28% harbored *ERBB2* fusions, which occurred more commonly in gastroesophageal junction cancer (3.12%; 3/96), breast cancer (1.89%; 8/422), urothelial carcinoma (1.72%; 1/58), and gastric cancer (1.60%; 23/1,437). Our population presented with a median age of 65 years (range 28 to 88 years), a high proportion of men (55 men vs 34 women; 61.80%). Among the patients with *ERBB2* fusions, *TP53* (82%), *APC* (18%), and *CDK4* (15%) were the top3 co-mutant genes. What’s more, most patients with *ERBB2* fusion also had *ERBB2* amplification (75.28%; 67/89), which was similar to the data in the TCGA database (88.00%; 44/50). Furthermore, TCGA database shows that patients with *ERBB2* fusions in pan-cancer had a worse prognosis than those without *ERBB2* fusions, as well as in breast cancer. Besides, *ERBB2* amplification combined with *ERBB2* fusion had worse prognosis than those with only *ERBB2* amplification. *ERBB2* fusion may interfere the effect of anti-HER2-targeted antibody drugs and influence the prognosis of patients with *ERBB2* amplification. Prospective clinical trials are warranted to confirm the results in the future.

## Introduction

Human epidermal growth factor receptor 2 (HER2), encoded by *ERBB2*, is an important member of the receptor tyrosine kinase (RTK) family. The HER2 receptor is activated by forming homodimers or heterodimers with other ERBB family receptors; particularly, it forms the most stable heterodimer with EGFR (1). Therefore, HER2 can enhance EGFR signaling and promote the continuous differentiation and proliferation of tumor cells (2). The oncogenic activation of HER2 can be caused by HER2 protein over-expression, gene amplification, or gene mutation and occur in various malignant tumors, including breast (3, 4), gastric (5), non-small cell lung (6), bladder (7), ovarian (8), and pancreatic cancers (9).

*ERBB2* amplification is the most common mechanism leading to increased HER2 protein over-expression. Among all the cancers related to *ERBB2* amplification and HER2 over-expression, breast cancer is most widely studied. *ERBB2*, amplified in 20%–30% of the breast cancer cases, is associated with aggressive tumor behavior (10). HER2 is also over-expressed in patients with other solid tumors, such as gastric cancer (11), biliary tract (12), colorectal (13), non-small cell lung (14), and bladder cancer (15).

Studies have suggested that the *ERBB2* mutations play an important role in the pathogenesis, development, and resistance to anti-HER2-targeted breast cancer drugs (16). *ERBB2* mutations are also found in other common cancers (17), including lung (18) and colorectal (19). *ERBB2*’s exon 20 mutations (4.83%) is a relatively frequent primary oncogenic driver in non-small cell lung cancer (NSCLC), especially lung adenocarcinoma (LUAD) (20). Therefore, anti-HER2 therapies such as trastuzumab, lapatinib, afatinib, and masatinib are effective in heavily pretreated *ERBB2*-mutated NSCLC (18).

In addition to amplification and SNV, there are various, albeit less common, fusion forms of *ERBB2*. For example, *ERBB2* fusions, representing a different mechanism of HER2 activation, have been described in gastric cancer. ZNF207-*ERBB2* and MDK-*ERBB2* fusion variants can activate HER2 signaling in a similar manner as wild-type HER2 (21). Meanwhile, *ERBB2* fusion has been found in colorectal cancer (22) and breast cancer (23), which may be related to response to HER2-targeted drugs. The mechanism of *ERBB2* fusion in tumors remains unclear and *ERBB2* fusion could be a potential target. Therefore, systematic research on *ERBB2* fusions has clinical significance. However, *ERBB2* fusions have not been systematically described in pan-cancer. Here, we retrospectively analyzed the genomic profiling of the *ERBB2* fusions from 32,131 Chinese patients with solid tumors. Our results comprehensively revealed enrichment of *ERBB2* fusions in certain histologic subtypes, providing a new insights of responses to therapies targeting *ERBB2*.

## Materials and method

### Patient information and sample collection

Between May 2018 and October 2021, 32,131 consecutive clinical samples of primarily relapsed and refractory solid tumors in the database of Simcere Diagnostics, Co. Ltd. (Nanjing, China) were evaluated retrospectively to search for *ERBB2* gene fusions. All the patients included in the study were informed consent concerning genetic testing and research. We identified patients with *ERBB2* fusions in the laboratory information management system (LIMS) database using a natural language search program. For those cases, relevant demographic and clinical data were extracted from the database, including age, gender, date of diagnosis, histology type, pathological stage, and evaluation of treatment responses per the reports of the clinical investigators. For tumor tissue samples, the pathologic diagnosis and tumor content for each case was confirmed by pathologists. We analyzed only those patients who had *ERBB2* fusion testing, among these cases, 66 cases were analyzed using the same panel containing 69 genes, which were used in co-occurring gene alterations analysis. Other patients chose some matched panels containing *ERBB2* fusion detection due to their conditions and needs.

### DNA extraction and library preparation

Three commercial kits were used for DNA extraction. Genomic DNA (gDNA) of formalin-fixed and paraffin-embedded (FFPE) tissues and fresh tissues was extracted using the Tissue sample DNA extraction kit (Kai Shuo). Genomic DNA of leucocytes was extracted using MagMAXTM DNA Multi-Sample Ultra Kit (Thermo). Cell-free DNA (cfDNA) of plasma was extracted using MagMAXTM Cell-Free DNA Isolation Kit (Thermo). All of the extraction procedures were performed following the manufacturer’s instructions. DNA was quantified on Qubit Fluorometer with Qubit dsDNA HS Assay kit (Thermo) and its quality was evaluated by Agilent 4200 TapeStation (Agilent).

The probe hybridization capture method was used for library construction. Commercial reagents and customized probes were used for library construction and hybridization capture. In brief, 15 ng-200 ng gDNA was sheared into 200~350 bp by fragmentation enzymes. Indexed paired-end adaptors for the Illumina platform were self-developed and customized (SimcereDx). End repair, A-tailing, and adaptor ligation of sheared DNA and cfDNA was respectively performed by KAPA HyperPlus DNA Library Prep kit (Roche Diagnostics) and VAHTSTM Universal DNA Library Prep Kit for Illumina^®^ (Vazyme). Unligated adaptors were removed by the size selection function of Agencourt AMPure XP beads (Beckman Coulter). The ligation products were PCR amplified to form a pre-library for hybridization. The final library was quantified on Qubit Fluorometer with Qubit dsDNA HS Assay kit (Thermo Fisher) and its quality was evaluated by Agilent 4200 TapeStation (Agilent).

### Sequence data processing

The qualified DNA libraries were sequenced on the Illumina NovaSeq6000 platform (Illumina, San Diego, CA) and generated 150 bp paired-end reads. Base calls from Illumina NovaSeq6000 were conducted to FASTQ files. The software fastp (v.2.20.0) was used for adapter trimming and filtering of low-quality bases (24). The BWA-MEM (v.0.7.17) algorithm was performed to align to the reference genome (UCSC’s hg19 GRCh37) (25). Duplicate reads from PCR were excluded using Dedup with Error Correct. SNVs/InDels were called and annotated *via* VarDict (v.1.5.7) (26) and InterVar (27), then the variants were filtered against the common SNPs in the public database including 1000 Genome Project (Aug 2015) and Exome Aggregation Consortium (ExAC) Browser28 (v.0.3). CNVs and fusions were analyzed by CNVkit (dx1.1) (28) and factera (v1.4.4) (29), respectively.

### Statistical analysis

The analysis of CNV difference between *ERBB2* amplification and *ERBB2* amplification combined with fusion using Wilcoxon test. All reported p-values were two-tailed, and p <0.05 was considered statistically significant. Statistical analyses were performed using R package ggpubr v. 0.4.0 (https://cran.r-project.org/package=ggpubr).

## Results

### Clinical characteristics of *ERBB2* fusion patients

**Figure 1:**
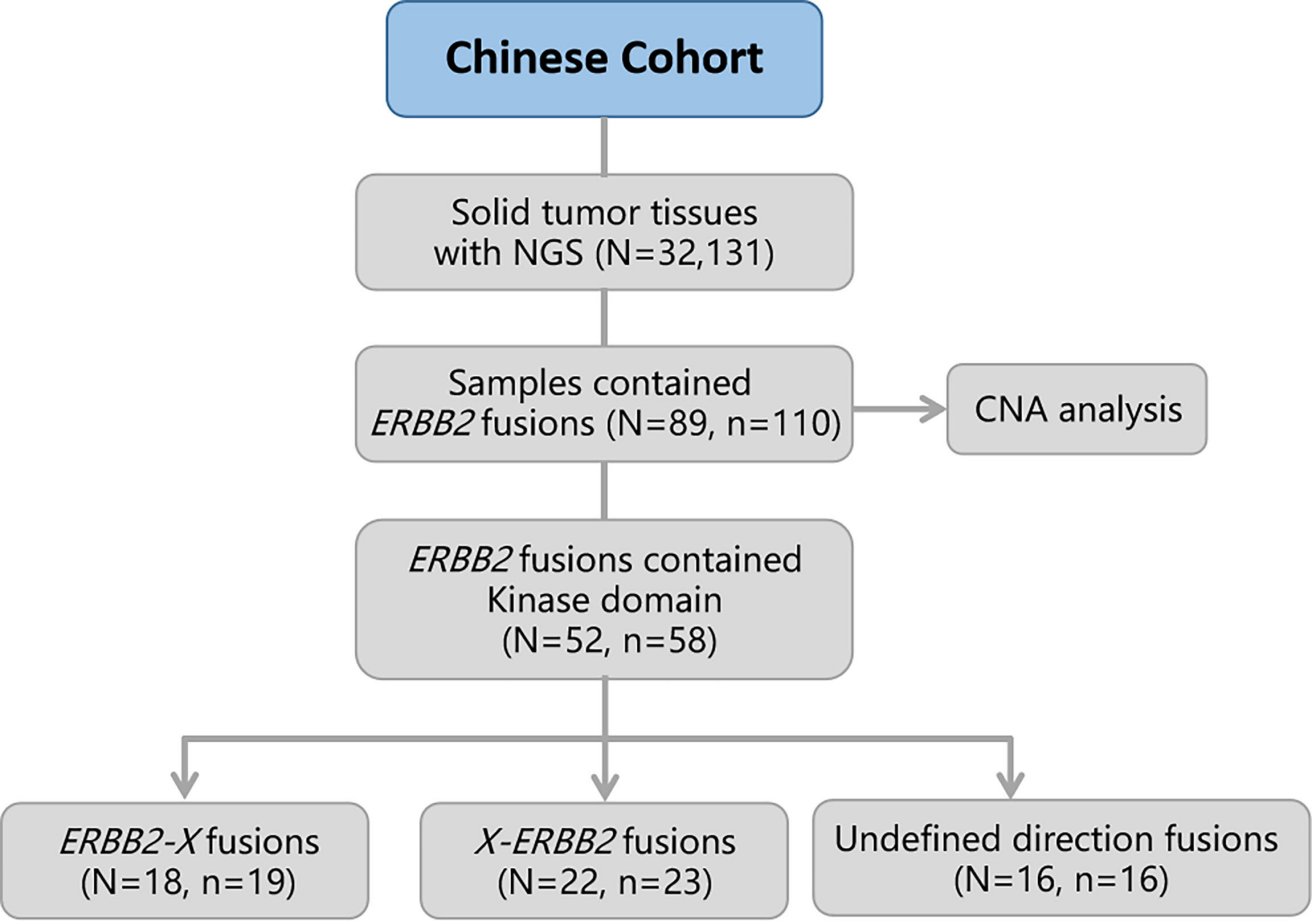
Flow diagram of the study. OS, overall survival; CNA, copy number alteration. N, number of patients; n, number of *ERBB2* fusions.

**Figure 2:**
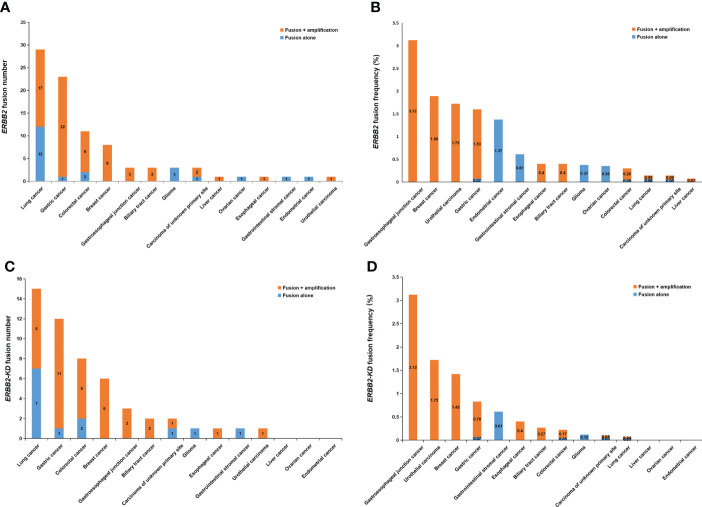
*ERBB2* fusions in solid tumors. **(A)** The number of *ERBB2* fusions in different cancer types. **(B)** The frequency of *ERBB2* fusions in different cancer types. **(C)** The number of *ERBB2-KD* fusions in different cancer types. **(D)** The frequency of *ERBB2-KD* fusions in different cancer types.

In total, eighty-nine (0.28%) of the 32,131 samples harbored 110 *ERBB2* fusions (**Figure 1**; **Table 1**). The median age of the 89 patients whose tumors harbored *ERBB2* fusions was 65 with a range of 28–88; 34 (38.20%) of the patients were female and 55 (61.80%) were male. Most patients had a clinical stage IV disease when initially diagnosed (**Table 1**). *ERBB2* fusions were distributed across different tumor types, including lung cancer (0.14%; 29/20,670), gastric cancer (1.60%; 23/1,437), colorectal cancer (0.30%; 11/3,613), breast cancer (1.89%; 8/422), gastroesophageal junction cancer (3.12%; 3/96), biliary tract cancer (0.40%; 3/745), glioma (0.37%; 3/814), liver cancer (0.07%; 1/1,406), ovarian cancer (0.35%, 1/285), esophageal cancer (0.40%; 1/249), gastrointestinal stromal tumor (0.61%; 1/163), endometrial cancer (1.37%, 1/73), urothelial carcinoma (1.72%; 1/58), and cancer with unknown primary sites (0.14%; 3/2100) (**Figures 2A, B** and **Table 2**). 75.28% (67/89) of the *ERBB2* fusion-positive patients also harbored *ERBB2* amplification, which was similar to 88.00% in TCGA cohort (**Supplementary Tables 1**, **2**).

**Table 1:** A summary of *ERBB2* fusion tumor patients’ demographic and clinical characteristics in Chinese cohort.

Characteristics	*ERBB2* fusions (N, %)
No. of patients	89
Gender	Female (34, 38.20%)
	Male (55, 61.80%)
Age (median, range)	65 (range: 28-88)
Diagnosis	Lung cancer (29, 32.58%)
	Gastric cancer (23, 25.85%)
	Colorectal cancer (11, 12.36%)
	Breast cancer (8, 8.99%)
	Others (18, 20.22%)
Pathologic stage	I (3, 3.37%)
	II (5, 5.62%)
	III (7, 7.87%)
	IV (26, 29.21%)
	Unknown (48, 53.93%)

**Table 2:** Frequencies of *ERBB2* alternations and relative distribution of alternation types in 89 *ERBB2* fusion solid tumors from Chinese cohort.

Cancer type	Samples	Fusion aloneN (%)	Fusion + amplificationN (%)	Fusion totalN (%)
Lung cancer	20,670	**12** (0.06)	**17** (0.08)	**29** (0.14)
Colorectal cancer	3,613	**2** (0.05)	**9** (0.25)	**11** (0.30)
Gastric cancer	1,437	**1** (0.07)	**22** (1.53)	**23** (1.60)
Liver cancer	1,406	0 (0)	**1** (0.07)	**1** (0.07)
Glioma	814	**3** (0.37)	0 (0)	**3** (0.37)
Biliary tract cancer	745	0 (0)	**3** (0.40)	**3** (0.40)
Breast cancer	422	0 (0)	**8** (1.89)	**8** (1.89)
Ovarian cancer	285	**1** (0.35)	0 (0)	**1** (0.35)
Esophageal cancer	249	0 (0)	**1** (0.40)	**1** (0.40)
Gastrointestinal stromal cancer	163	**1** (0.61)	0 (0)	**1** (0.61)
Gastroesophageal junction cancer	96	0 (0)	**3** (3.12)	**3** (3.12)
Endometrial cancer	73	**1** (1.37)	0 (0)	**1** (1.37)
Urothelial carcinoma	58	0 (0)	**1** (1.72)	**1** (1.72)
Carcinoma of unknown primary site	2100	**1** (0.05)	**2** (0.09)	**3** (0.14)
Total	32,131	**22** (0.07)	**67** (0.21)	**89** (0.28)

The bolded values/numbers mean that ERBB2 fusions were detected in ≥1 patient in the corresponding cancer type.

Considering the characteristics of the receptor tyrosine kinase, we focused on the *ERBB2* fusions which contain the kinase domain. Fifty-two of which can produce an intact HER2 kinase domain (*ERBB2-KD* fusion) and were collected to conduct follow-up analysis (**Figure 1**). The median age of the 52 patients whose tumors harbored *ERBB2-KD* fusions was 63 with a range of 28–88; 22 (42.31%) of the patients were female and 30 (57.69%) were male. Most patients had a clinical stage IV disease when initially diagnosed (**Table 3**). *ERBB2-KD* fusions occurred more commonly in gastroesophageal junction cancer (3.12%; 3/96), urothelial carcinoma (1.72%; 1/58), breast cancer (1.42%; 6/422), and gastric cancer (0.83%; 12/1,437) (**Figures 2C, D** and **Table 4**).

**Table 3:** A summary of *ERBB2-KD* fusion tumor patients’ demographic and clinical characteristics in Chinese cohort.

Characteristics	*ERBB2-KD* fusions (N, %)
No. of patients	52
Gender	Female (22, 42.31%)
	Male (30, 57.69%)
Age (median, range)	63 (range: 28-88)
Diagnosis	Lung cancer (15, 28.85%)
	Gastric cancer (12, 23.08%)
	Colorectal cancer (8, 15.38%)
	Breast cancer (6, 11.54%)
	Others (11, 21.15%)
Pathologic stage	I (3, 5.77%)
	II (3, 5.77%)
	III (3, 5.77%)
	IV (15, 28.84%)
	Unknown (28, 53.85%)

**Table 4:** Frequencies of *ERBB2* alternations and relative distribution of alternation types in 52 *ERBB2-KD* fusion solid tumors from Chinese cohort.

Cancer type	Samples	Fusion aloneN (%)	Fusion + amplificationN (%)	Fusion totalN (%)
Lung cancer	20,670	**7** (0.03)	**8** (0.04)	**15** (0.07)
Colorectal cancer	3,613	**2** (0.05)	**6** (0.17)	**8** (0.22)
Gastric cancer	1,437	**1** (0.07)	**11** (0.76)	**12** (0.83)
Liver cancer	1,406	0 (0)	0 (0)	0 (0)
Glioma	814	**1** (0.12)	0 (0)	**1** (0.12)
Biliary tract cancer	745	0 (0)	**2** (0.27)	**2** (0.27)
Breast cancer	422	0 (0)	**6** (1.42)	**6** (1.42)
Ovarian cancer	285	0 (0)	0 (0)	0 (0)
Esophageal cancer	249	0 (0)	**1** (0.40)	**1** (0.40)
Gastrointestinal stromal cancer	163	**1** (0.61)	0 (0)	**1** (0.61)
Gastroesophageal junction cancer	96	0 (0)	**3** (3.12)	**3** (3.12)
Endometrial cancer	73	0 (0)	0 (0)	0 (0)
Urothelial carcinoma	58	0 (0)	**1** (1.72)	**1** (1.72)
Carcinoma of unknown primary site	2100	**1** (0.05)	**1** (0.05)	**2** (0.10)
Total	32,131	**13** (0.04)	**39** (0.12)	**52** (0.16)

Samples: total number of patients, fusion alone: number of ERBB2-KD pure fusion samples, fusion + amplification: number of ERBB2-KD fusion combined with ERBB2 amplification samples, fusion total: number of total ERBB2-KD fusion samples. The bolded values/numbers mean that ERBB2 fusions were detected in ≥1 patient in the corresponding cancer type.

### Identification of *ERBB2-KD* fusion partners

**Figure 3:**
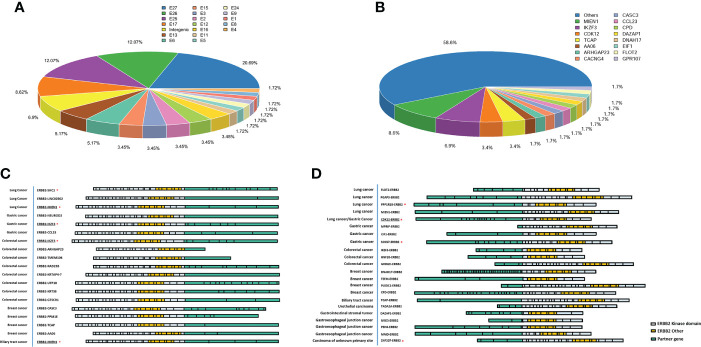
*ERBB2-KD* rearrangements in solid tumors. **(A)** Frequency of fusion between different exons of *ERBB2*. **(B)** Frequency of *ERBB2-KD* fusion variants’ partners. Schematic of all *ERBB2-X* fusions **(C)** and *X-ERBB2* fusions **(D)** identified, at scale with exons represented by individual boxes. The partners is colored pink, with *ERBB2* kinase domain colored orange and other *ERBB2* exons colored white. The reported *ERBB2* fusions marked with the red asterisk, others were novel *ERBB2* fusions. The underlined marks are the same fusion gene with different break point positions.

Of the exon composition of the *ERBB2-KD* fusions identified, all 58 *ERBB2-KD* fusions from 52 patients contained intact *ERBB2* kinase domain encoded by exons 18–24 (**Supplementary Materials**). Fusion of *ERBB2* gene was more common in exons 17, 25, 26, and 27 (**Figure 3A**). Two breast cancer patients and two colorectal cancer patients had 2 different *ERBB2-KD* fusions each, and 1 colorectal cancer patient had 3 different *ERBB2-KD* fusions. The remaining 47 patients had one *ERBB2-KD* fusion each. *MIEN1* was the most frequent fusion partner identified in 5 cases, followed by *IKZF3* with 4 fusions, and *CDK12* and *TCAP* with 2 fusions. All the other fusions were identified in only 1 case (**Figure 3B**).

Consider the binding mechanism of HER2-targeted drugs, we divided the *ERBB2-KD* fusion into two classes for analysis. Those *ERBB2-X* fusions which were intact from the extracellular domain to the kinase domain, while those X-*ERBB2* fusions only retained kinase domain. In our study, 19 *ERBB2-X* and 23 *X-ERBB2* fusions were identified (**Figures 3C, D**), and the other *ERBB2-KD* fusions’ direction was uncertain. In *ERBB2-X* fusions (**Figure 3C**), there were 17 different fusion partners, of which 3 have been previously reported, and 14 were previously unreported in public databases (COSMIC and TCGA) or published literature (PubMed). In addition, the *ERBB2-IKZF3* fusion was appeared in two patients each, the same as the *ERBB2-MIEN1* fusion. The 23 *X-ERBB2* fusions detected from 22 patients all could encode an intact HER2 kinase domain. One breast cancer patient had 2 different *ERBB2-KD* fusions, and the remaining 21 patients had 1 *ERBB2-KD* fusion. Two *CDK12-ERBB2* fusions were identified in gastric tumor and lung cancer. All the other fusions were recognized in only one case. The *X-ERBB2* fusions had 22 different fusion partners, of which 4 have been previously reported, and 18 were novel (**Figure 3D**). All 23 *ERBB2* fusions were in-frame with breakpoints that were distributed in different introns and exons of *ERBB2* (**Supplementary Table 3**).

### Co-occurring gene alterations

**Figure 4:**
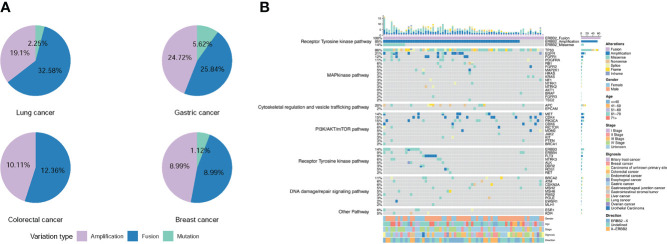
Frequency and distribution of *ERBB2* variations in solid tumors with *ERBB2* fusion. **(A)** Distribution of *ERBB2* variations in three cancer types with *ERBB2* fusion, lung cancer, gastric cancer, colorectal cancer, and breast cancer. For each cancer type, the frequency of *ERBB2* variations was described as the percentage of all cancer cases analyzed, and the distribution and type of variations were normalized to 100%. **(B)** Landscape of genomic aberrations of *ERBB2* fusion in solid tumors.

Many patients with *ERBB2* fusions also had *ERBB2* amplification (75.28%; 67/89) and *ERBB2* SNV (8.99%; 8/89). In contrast, patients with only *ERBB2* fusions were less common (24.72%; 22/89) (**Table 2** and **Figure 4A**). *ERBB2* fusion combined with amplification was found in patients with lung, colorectal, gastric, liver, biliary tract, breast, esophageal, and gastroesophageal junction cancers and urothelial carcinoma. *ERBB2* fusion alone was found in patients with lung, colorectal, gastric, glioma, ovarian, endometrial, and gastrointestinal stromal tumors.

We then analyzed the co-mutations of 69 tumor driver genes (listed in the **Supplementary Table 4**) with *ERBB2* in 66 patients with *ERBB2* fusions. The top 5 co-mutated genes included *TP53* (86%), *EGFR* (21%), *APC* (20%), *CDK4* (15%), *PIK3CA* (15%) (**Figure 4B**). Among them, *TP53* and *PIK3CA* were mainly concentrated on missense mutations, *EGFR* and *CDK4* were mainly amplification, while *APC* was missense and nonsense mutations. In the *ERBB2* fusion alone, the co-mutated genes were mainly missense mutations of *TP53*, *KRAS*, *NF1*, *BRAF*, *APC*, *PTEN*, *ERBB4*, *ROS1* and amplifications of *FGFR2*, *MET*, *CDK4*, and *CDK6*. The most concurrently-mutated genes with *ERBB2* fusions were in the MAP kinase, RTK, cytoskeletal regulation and vesicle trafficking, PI3K/Akt/mTOR, and DNA damage/repair signaling pathways.

### CNV difference between *ERBB2* amplification and *ERBB2* amplification combined with fusion

**Figure 5:**
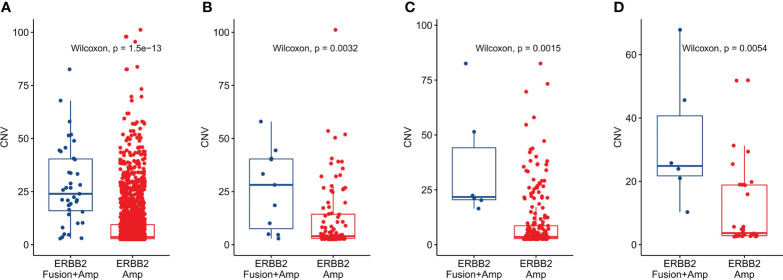
CNV difference between solid tumors with only *ERBB2* amplification and tumors with *ERBB2* fusion combined with amplification. **(A)** CNV difference analysis classified by *ERBB2* fusion status in pan-cancer, **(B)** CNV difference analysis classified by *ERBB2* fusion status in gastric cancer. **(C)** CNV difference analysis classified by *ERBB2* fusion status in colorectal cancer. **(D)** CNV difference analysis classified by *ERBB2* fusion status in breast cancer.

Since more than 75% of *ERBB2* fusions were accompanied by *ERBB2* amplification, we also analyzed the difference in CNV values between the pure *ERBB2* amplification (*ERBB2* Amp) and *ERBB2* amplification combined with fusion (*ERBB2* Fusion + Amp) in different cancer types. At the same time, since *ERBB2* amplification mainly occurs in gastric cancer, colorectal cancer, and breast cancer, we focused on these cancer types in our work. The results showed that the CNV of *ERBB2* Amp was significantly higher than *ERBB2* Fusion + Amp in pan-cancer (**Figure 5A**), gastric cancer (**Figure 5B**), colorectal cancer (**Figure 5C**), and breast cancer (**Figure 5D**) (P<0.01).

## Discussion

*ERBB2* is one of the most common oncogenic driver genes; its amplification alterations predominantly occur in about 30% of breast cancer (30) and 20% of gastric cancer cases (31). Previously, most studies focused on *ERBB2* amplifications. Therefore, there is a need for comprehensive studies on other alterations of *ERBB2*, such as *ERBB2* fusions.

This study identified 110 *ERBB2* fusions from 32,131 samples with solid tumors using NGS. Although *ERBB2* fusions were rare, they occurred more frequently in gastroesophageal junction cancer (3.12%), breast cancer (1.89%), and urothelial carcinoma (1.72%). Considering the characteristics of the receptor tyrosine kinase, *ERBB2* fusions containing the kinase domain were screened. There were 58 *ERBB2* fusions containing a full kinase domain (*ERBB2-KD* fusions) in 52 patients. The *ERBB2-KD* fusion partners were diverse. Among the 42 fusions with certain directions, 32 fusions were first reported by us. *TP53*, *EGFR*, *APC*, *CDK4*, and *PI3KCA* were relatively frequent mutant genes in patients with *ERBB2* fusions, and most of the concurrently-mutated genes were identified in the RTK, MAP Kinase, DNA damage/repair signaling, PI3K/Akt/mTOR, and cell cycle pathways. Interestingly, we found that most *ERBB2* fusions accompanied *ERBB2* amplification, which was the main target of anti-HER2 therapy, while *ERBB2* fusions rarely occur by themselves.

The HER2-targeting drugs were divided into two categories, trastuzumab, pertuzumab, and TDM-1, which binding to the proximal membrane domain of HER2, while lapatinib and neratinib binding to the intracellular kinase domain. Therefore, we distinguished the different fusions by the position of *ERBB2* in the fusion protein and divided them into *ERBB2-X* fusions and *X-ERBB2* fusions. Given the presence of the HER2 dimerization domain (32, 33), all of *ERBB2-X* and *X-ERBB2* fusion variants were likely to form homodimers in a manner similar to that of amplified wild-type HER2. Meanwhile, they all retained the kinase domain. Therefore, we speculated that these fusions were meaningful. The *ERBB2-X* fusions retained the intact extracellular domain and can bind to ligands and monoclonal antibodies theoretically. Patients with these *ERBB2-X* fusions might benefit from HER2 antibodies therapy. While *X-ERBB2* fusion proteins, due to the deletion of the signal peptide and transmembrane sequence, were most likely retained in the cytoplasm, which might be effective with HER2-TKIs and might also lead to HER2 antibodies’ resistance in patients with *ERBB2* amplification.

As described in the result, most *ERBB2* fusions accompanied *ERBB2* amplification. The current clinical protocol for selecting HER2-positive patients is based on FISH positivity or an IHC score of 3+, while an IHC score of 2+ requires confirmation using FISH. The FISH probe covers the entire *ERBB2* (34). Meanwhile, the antibody used in IHC recognizes the HER2 epitope located at HER2’s intracellular site (35–37); it also covers all *X-ERBB2* fusion proteins in this study. Therefore, only the special probes design for detection fusions can distinguish the *ERBB2* amplification and fusion by FISH, but NGS could be a feasible, high-throughput method to detect *ERBB2* fusions.

A limitation of this study was the lack of a large sample of *ERBB2* fusion patients’ prognosis in the real world. However, given the importance of clinical prognosis, we attempted a simple exploratory prognostic analysis of *ERBB2* fusions in the TCGA database. The results indicated that the prognosis of the patients with *ERBB2* fusions was significantly worse than those without *ERBB2* fusions in pan-cancer and breast cancer (**Supplementary Figures 1A, B**). Besides, the prognosis of these patients harbored *ERBB2* amplification only was better than those with *ERBB2* fusions combination with amplification in pan-cancer (**Supplementary Figure 1C**). In addition, in breast cancer, the OS of the patients with *ERBB2* amplification was significantly longer than those with *ERBB2* fusions with amplification (**Supplementary Figure 1D**). Since there was no specific CNV value in TCGA cohort, we could not compare the difference of CNV between the two groups. The CNV was significantly higher in *ERBB2* amplification combined with fusion than pure *ERBB2* amplification in Chinese cohort, we speculate that this phenomenon may also exists in TCGA cohort. High CNV had been revealed as an independent risk factor predicted unfavorable prognosis in HER2-positive gastric adenocarcinoma patients (38), and HER2-positive metastatic breast cancer patients with high HER2 CNV in plasma had worse prognosis after trastuzumab-based therapy (39). Based on our founding and other study results, there were two possibilities. On one hand, the impact of *ERBB2* fusions on clinical outcomes might be caused by high CNV. On the other hand, the impact of high CNV on clinical outcomes might be due to the presence of *ERBB2* fusions, or some other possibility. Which one is right? We do not have a definite answer to this question at present, and further basic research and mechanism research are needed.

The important thing is that *ERBB2* fusion seems not meaningless “passenger fusion” without function. Previous *in vitro* studies showed that the cells expressing *ZNF207 (exon 2)-ERBB2 (exon18)* fusion gene lost the ability to bind to T-DM1, which is trastuzumab conjugated with emtansine (DM1), an antimitotic agent. Furthermore, *in vivo* efficacy study indicated that trastuzumab did not inhibit tumor growth in xenografts expressing the ZNF207-ERBB2 fusion; this finding supported the resistant mechanism to trastuzumab (21). ZNF207 is a kinetochore- and microtubule-binding protein that plays a key role in spindle assembly. It localize in cytoskeleton, kinetochore, and nucleus. We speculate that since this protein does not have a signal peptide, when it is fused with HER2, this ZNF207-HER2 fusion protein cannot localize on the cell membrane, so it is not sensitive to trastuzumab or T-DM1, instead the HER2-TKIs may be effective.

Regarding the effect of *ERBB2* fusions on the efficacy of targeted drugs, there are also related reports on colorectal cancer and breast cancer. For example, in patients with colorectal carcinoma, ERBB2-GRB7 fusion is insensitive to HER2 inhibitors (22). Lesurf et al. (23) retrospectively analyzed the correlation between the molecular characteristics of 48 patients in the clinical trial of ACOSOG Z1041 and the efficacy of trastuzumab combined with chemotherapy, i.e., neoadjuvant therapy, for breast cancer and found that 2 patients with *ERBB2* fusions (*ERBB2* (exon19)-IKZF3, *ERBB2* (exon1)-TBC1D3P1-DHX40P1) did not achieve pathologic complete response (pCR). HER2-TBC1D3P1-DHX40P1 only retains one exon region of HER2, so it is not sensitive to targeted drugs. The kinase domain of HER2 protein covers exon 18 to exon 24, while HER2-IKZF3 fusion proteins do not retain the complete kinase domain, and we speculate that this may be the reason for the patient insensitive to trastuzumab. Therefore, we conclude that *ERBB2* fusion partners and breakpoint locations play a vital role in HER2-targeted therapy.

In addition to antibody drugs, several small-molecule HER2 kinase inhibitors, including pyrotinib and lapatinib, are available approved agents for patients with HER2-positive breast cancer. Besides, pyrotinib has demonstrated a good potential to treat advanced NSCLC with *ERBB2* mutations, especially the *ERBB2* exon 20 insertions, in the phase II studies (40, 41). In view of their drug mechanisms which bind to the HER2 kinase domain, pyrotinib or lapatinib can be a potential option for cancer patients with *ERBB2* fusions, especially those *X-ERBB2* fusions, which needs to be prospectively explored.

Our study is the first comprehensive analysis of a large group of Chinese patients with pan-cancer, it has extended our understanding of *ERBB2* fusions in solid tumors significantly. However, there were several limitations in our study. First, we couldn’t collect complete and detailed clinical pathological characteristics and treatment details including survival status of all patients with *ERBB2* fusion. To analyze the prognosis of *ERBB2* fusion, we utilized the TCGA database and found patients with *ERBB2* fusion had a worse prognosis than those without *ERBB2* fusion. In addition, the patients harboring *ERBB2* fusion and *ERBB2* amplification showed a worse prognosis than patients with pure *ERBB2* amplification, or *ERBB2* amplification without fusion. Second, the function of this novel *ERBB2* fusion proteins and the potential effect of *ERBB2* fusion to anti-HER2 therapy have not been studied and described clearly. By analyzing the protein structure and functional sequence of HER2, we screened out some potentially meaningful *ERBB2* fusion forms. Basic experiments carrying out are exploring the function of the fusion protein, followed by responsiveness to various anti-HER2 drugs. Despite its limitations, this study is the first comprehensive analysis of *ERBB2* fusions in a larger group of Chinese patients with various carcinomas, it has extended our understanding of *ERBB2* fusions in solid tumors significantly and may provide more inspiration in the upcoming clinical trials.

## Conclusions

This study analyzed the *ERBB2* fusions profiles in more than 30,000 patients with different solid carcinomas. The prevalence of this rare mutation differs obviously and is relatively higher in upper digestive system tumor. Most *ERBB2* fusions are accompanied by *ERBB2* amplification and may play a role in the prognosis of pan-cancer, especially BRCA. Further well-designed prospective researches are expected to confirm the role of *ERBB2* fusion and to identify the patients who will benefit more from anti-HER2 treatment.

## Data availability statement

The original contributions presented in the study are included in the article/**Supplementary Material**. Further inquiries can be directed to the corresponding authors.

## Ethics statement

The studies involving human participants were reviewed and approved by Beijing Chao-Yang Hospital, Capital Medical University. The patients/participants provided their written informed consent to participate in this study.

## Author contributions

YG and YF performed the research, collected and analyzed the data, interpreted the results and helped revise the manuscript. YW, HL, and JM prepared samples, gathered detailed clinical information for the study. XY collected and analyzed the data, and wrote the initial draft of the paper. XZ and QZ were responsible for bioinformatics investigation. TS and CQ were responsible for statistical review and participated in review. GA and BX conceived the study, directed and supervised research, and revised the manuscript. The work reported in the paper has been performed by the authors unless clearly specified in the text. All authors contributed to the article and approved the submitted version.

## Acknowledgments

We appreciate the effort of the physicians for enrolling patients and thank all the patients involved for allowing us to analyze their clinical data. Besides, we are grateful to Qianru He and Qianqian Duan for their help in the study.

## Conflict of interest

Authors XY, QZ, XZ, TS, and CQ were employed by Jiangsu Simcere Diagnostics Co., Ltd, Nanjing Simcere Medical Laboratory Science Co., Ltd.

The remaining authors declare that the research was conducted in the absence of any commercial or financial relationships that could be construed as a potential conflict of interest.

## Publisher’s note

All claims expressed in this article are solely those of the authors and do not necessarily represent those of their affiliated organizations, or those of the publisher, the editors and the reviewers. Any product that may be evaluated in this article, or claim that may be made by its manufacturer, is not guaranteed or endorsed by the publisher.

## Abbreviations

Amp, Amplification; BRCA, Breast invasive carcinoma; CNV, Copy number variation; FISH, Fluorescent *in situ* hybridization; HER2, Human epidermal growth factor receptor 2; IHC, Immunohistochemical; KD, Kinase domain; MAP kinase, Mitogen Activated Protein Kinases; NGS, Next-generation sequencing;NSCLC, Non-Small Cell Lung Cancer; OS, Overall survival; PD, Progressive disease;RTK, Receptor Tyrosine Kinase; SNV, Single-nucleotide variant; TKI, Tyrosine kinase inhibitors; T-DM1, Trastuzumab emtansine
