# Supplementary material for: Molecular and clinicopathological characteristics of ERBB2 gene fusions in 32,131 Chinese patients with solid tumors
Source: Front Oncol. 2022 Oct 6;12:986674. doi: 10.3389/fonc.2022.986674 (PMC9582139; doi:10.3389/fonc.2022.986674)
Supplement: Supplementary file 1 [file DataSheet_1.docx]

Supplementary Material

# Supplementary Materials and Method

The Cancer Genome Atlas (TCGA) cohort

All clinical and genomic data of 9678 solid tumors across 21 tumor types were retrieved from cBioPortal (www.cbioportal.org). Those data were collected from The Cancer Genome Atlas (TCGA) Pan-Cancer analysis project (1). The copy number variation (CNV) pipeline and pipelines for the detection of gene fusions were described on the GDC documentation website (https://docs.gdc.cancer.gov/Data/Introduction/).

Statistical analysis

The Kaplan-Meier curve analysis OS was compared using the log-rank test. All reported P values were two-tailed, and P < 0.05 was considered statistically significant. Statistical analyses were performed using R v. 4.0.3 (https://www.r-project.org).

# Supplementary Figures and Tables

## Supplementary Figure

##
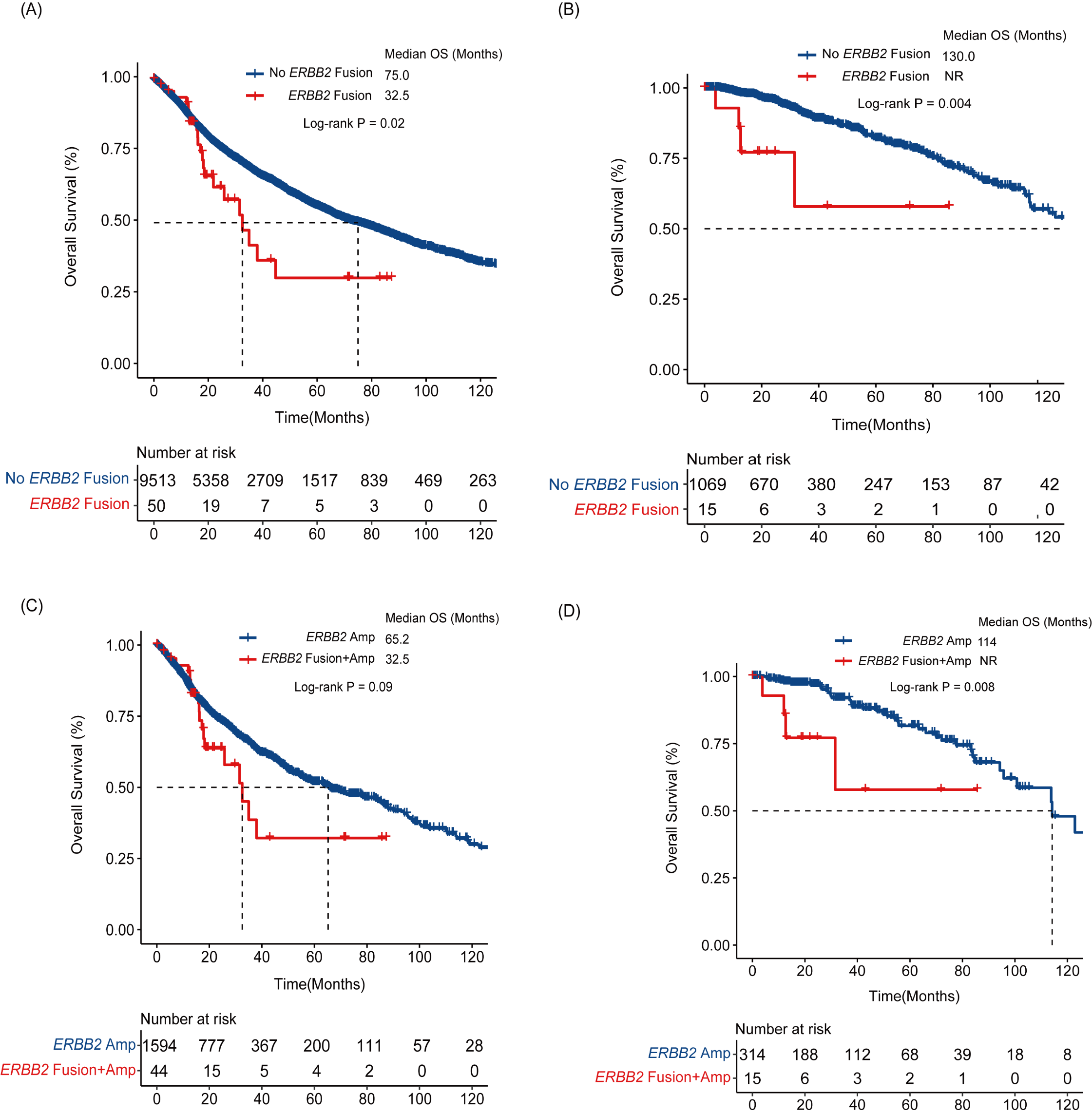


**Supplementary Figure 1.** Association of *ERBB2* different variants with Prognosis in TCGA Cohort. Kaplan-Meier survival analysis classified by *ERBB2* fusion status in pan-cancer (A), Kaplan-Meier survival analysis classified by *ERBB2* fusion status in BRCA (B). Kaplan-Meier survival analysis classified by *ERBB2* fusion status in pan-cancer with *ERBB2* amplification (C), Kaplan-Meier survival analysis classified by *ERBB2* fusion status in BRCA with *ERBB2* amplification (D).

## Supplementary Tables

**Supplementary Table 1** Frequencies of *ERBB2* alternations and relative distribution of alternation types in solid tumors from TCGA cohort

| **Cancer type** | **Samples** | **Fusion alone**  **N (%)** | **Fusion + amplification**  **N (%)** | **Fusion + deletion**  **N (%)** | **Fusion total**  **N (%)** |
| --- | --- | --- | --- | --- | --- |
| BRCA | 1,084 | 0 (0) | **15** (1.38) | 0 (0) | **15** (1.38) |
| COAD | 594 | 0 (0) | **1** (0.17) | 0 (0) | **1** (0.17) |
| OV | 585 | 0 (0) | **1** (0.17) | **1** (0.17) | **2** (0.34) |
| GBM | 585 | 0 (0) | 0 (0) | 0 (0) | 0 (0) |
| LUAD | 566 | 0 (0) | **1** (0.18) | 0 (0) | **1** (0.18) |
| UCEC | 529 | 0 (0) | **3** (0.57) | 0 (0) | **3** (0.57) |
| HNSC | 523 | 0 (0) | **2** (0.38) | 0 (0) | **2** (0.38) |
| LGG | 514 | 0 (0) | 0 (0) | 0 (0) | 0 (0) |
| KIRC | 512 | 0 (0) | 0 (0) | 0 (0) | 0 (0) |
| THCA | 499 | 0 (0) | 0 (0) | 0 (0) | 0 (0) |
| PRAD | 494 | 0 (0) | 0 (0) | 0 (0) | 0 (0) |
| LUSC | 487 | 0 (0) | **5** (1.03) | 0 (0) | **5** (1.03) |
| SKCM | 442 | 0 (0) | 0 (0) | 0 (0) | 0 (0) |
| STAD | 440 | 0 (0) | **5** (1.14) | 0 (0) | **5** (1.14) |
| BLCA | 411 | **2** (0.49) | **3** (0.73) | 0 (0) | **5** (1.22) |
| LIHC | 372 | 0 (0) | 0 (0) | **1** (0.27) | **1** (0.27) |
| CESC | 297 | 0 (0) | **3** (1.01) | **1** (0.34) | **4** (1.35) |
| SARC | 255 | 0 (0) | 0 (0) | 0 (0) | 0 (0) |
| PAAD | 184 | 0 (0) | **2** (1.09) | 0 (0) | **2** (1.09) |
| ESCA | 182 | **1** (0.55) | **3** (1.65) | 0 (0) | **4** (2.20) |
| THYM | 123 | 0 (0) | 0 (0) | 0 (0) | 0 (0) |
| Total | 9,678 | **3** (0.03) | **44** (0.45) | **3** (0.03) | **50** (0.52) |

Samples: total number of patients, fusion alone: number of *ERBB2* pure fusion samples, fusion + amplification: number of *ERBB2* fusion combined with amplification samples, fusion + deletion: number of *ERBB2* fusion combined with *ERBB2* deletion samples, fusion total: number of total *ERBB2* fusion samples.

Abbreviations: BRCA: Breast invasive carcinoma, COAD: Colon adenocarcinoma, OV: Ovarian serous cystadenocarcinoma, GBM: Glioblastoma multiforme, LUAD: Lung adenocarcinoma, UCEC: Uterine Corpus Endometrial Carcinoma, HNSC: Head and Neck squamous cell carcinoma, LGG: Brain Lower Grade Glioma, KIRC: Kidney renal clear cell carcinoma, THCA: Thyroid carcinoma, PRAD: Prostate adenocarcinoma, LUSC: Lung squamous cell carcinoma, SKCM: Skin Cutaneous Melanoma, STAD: Stomach adenocarcinoma, BLCA: Bladder Urothelial Carcinoma, LIHC: Liver hepatocellular carcinoma, CESC: Cervical squamous cell carcinoma and endocervical adenocarcinoma, SARC: Sarcoma, PAAD: Prostate adenocarcinoma, ESCA: Esophageal carcinoma, THYM: Thymoma.

**Supplementary Table 2 *ERBB2* amplification in *ERBB2* fusion patients**

| ***ERBB2* fusion/amplification** | **Frequency (%)** |
| --- | --- |
| ***ERBB2* CNV** |  |
| Gain | 44 (88%) |
| Loss | 3 (6%) |
| Normal | 3 (6%) |
| ***ERBB2* Partner** |  |
| *PPP1R1B* | 10 (20%) |
| *CTTN* | 8 (16%) |
| *IKZF3* | 5 (10%) |
| *PSMB3* | 3 (6%) |
| WIPF2 | 2 (4%) |
| Others | 22 (44%) |

# References

1. Cancer Genome Atlas Research Network, Weinstein JN, Collisson EA, Mills GB, Shaw KR, Ozenberger BA, et al. The Cancer Genome Atlas Pan-Cancer analysis project. *Nat Genet* (2013)45: 1113–1120. doi: 10.1038/ng.2764.
